# Targeting endogenous proteins for degradation through the affinity-directed protein missile system

**DOI:** 10.1098/rsob.170066

**Published:** 2017-05-10

**Authors:** Luke J. Fulcher, Luke D. Hutchinson, Thomas J. Macartney, Craig Turnbull, Gopal P. Sapkota

**Affiliations:** Medical Research Council Protein Phosphorylation and Ubiquitylation Unit, Dundee, UK

**Keywords:** proteolysis, AdPROM, ubiquitination, SHP2, ASC, nanobody

## Abstract

Targeted proteolysis of endogenous proteins is desirable as a research toolkit and in therapeutics. CRISPR/Cas9-mediated gene knockouts are irreversible and often not feasible for many genes. Similarly, RNA interference approaches necessitate prolonged treatments, can lead to incomplete knockdowns and are often associated with off-target effects. Targeted proteolysis can overcome these limitations. In this report, we describe an affinity-directed protein missile (AdPROM) system that harbours the von Hippel–Lindau (VHL) protein, the substrate receptor of the Cullin2 (CUL2) E3 ligase complex, tethered to polypeptide binders that selectively bind and recruit endogenous target proteins to the CUL2-E3 ligase complex for ubiquitination and proteasomal degradation. By using synthetic monobodies that selectively bind the protein tyrosine phosphatase SHP2 and a camelid-derived VHH nanobody that selectively binds the human ASC protein, we demonstrate highly efficient AdPROM-mediated degradation of endogenous SHP2 and ASC in human cell lines. We show that AdPROM-mediated loss of SHP2 in cells impacts SHP2 biology. This study demonstrates for the first time that small polypeptide binders that selectively recognize endogenous target proteins can be exploited for AdPROM-mediated destruction of the target proteins.

## 1. Introduction

Achieving targeted proteolysis of endogenous cellular proteins is not only desirable for research into their function, but is increasingly sought after as an approach to disrupt target protein function in therapeutics. The ubiquitin–proteasome system (UPS) is a major player for the proteolysis of many cellular proteins for maintenance of homeostasis [1–3]. Through sequential actions of the E1 ubiquitin-activating enzyme, E2 ubiquitin-conjugating enzymes and E3 ubiquitin ligases, target proteins are covalently labelled with ubiquitin chains, marking them for recognition and destruction by the proteasome [4,5]. The Cullin RING (really interesting new gene) E3 ligase (CRL) family represents one of the key contributors to the UPS for protein turnover in cells [6,7]. Each CRL complex comprises one Cullin (CUL1-7) member, one or more adaptors that bind to a substrate receptor and a RING E3 ligase (RBX1/2) [6,7]. For example, the CUL2-CRL is in a complex with Elongin B and C as adaptors, the von Hippel–Lindau (VHL) protein as the substrate receptor, and RING-box protein 1 (RBX1) as the E3 ligase [8,9]. All CRLs are activated through the covalent attachment of the small ubiquitin-like modifier NEDD8 to lysine residues of the Cullin (a process termed NEDDylation [10]), and the pan-Cullin NEDDylation inhibitor MLN4924 inhibits CRL activation [11].

By combining the CUL2 machinery and anti-GFP nanobodies (aGFP), we recently reported an efficient affinity-directed protein missile (AdPROM) system for proteolysis of endogenous target proteins that were marked by knocking in a GFP-tag using CRISPR/Cas9. This AdPROM system was engineered with the CUL2 substrate receptor VHL tethered to aGFP [12]. The efficacy of this proteolytic AdPROM suggested that this technology could be applied to achieve degradation of endogenous proteins by simply replacing the aGFP with small polypeptide binders that specifically recognize endogenous target proteins. Recent advances in polypeptide binder technologies have greatly expedited the development of highly selective, high-affinity polypeptide interactors of proteins [13,14]. One approach frequently employed to derive such polypeptide binders involves isolating single domain VHH antibodies (nanobodies) from camelid species, such as llama and alpaca, after immunization with protein antigens [15]. Nanobodies recognizing GFP and the human protein Apoptosis-associated speck-like protein containing CARD (ASC) are examples of camelid-derived nanobodies [15,16]. A second method exploits a synthetic approach, typically using a β-sandwich protein based on the 10th human fibronectin type III domain as a backbone, where varying combinations of residues in the variable regions are screened for high-affinity interactions with target proteins. These are referred to as monobodies [14]. A series of monobodies selectively recognizing the Src-homology 2 (SH2) domain-containing phosphatase 2 (SHP2) (also known as *PTPN11*) and the non-receptor tyrosine protein kinase Abl were developed using this synthetic screening approach [17,18].

In this study, we explore the efficacy of the AdPROM system to degrade endogenous SHP2 and ASC across different cell lines using their cognate mono/nanobodies as affinity probes. SHP2 is a non-receptor tyrosine phosphatase that is ubiquitously expressed in vertebrate cells and tissues [19]. SHP2 possesses two SH2 domains, termed N-SH2 and C-SH2, and a protein tyrosine phosphatase (PTP) domain. Activating mutations in SHP2 have been reported in multiple pathologies, including Noonan syndrome, juvenile myelomonocytic leukaemia, and lung and breast cancers [20]. SHP2 is reported to play a major role in mediating Ras/MAPK signalling, although this role appears to be context-specific [21]. The precise role of SHP2 phosphatase activity in MAPK signalling is not fully elucidated, in part because very few substrates of SHP2 are known. SHP2 has been reported to mediate transformation of cells, and modulate MAPK and signal transducer and activator of transcription 3/5 (STAT3/5), signalling downstream of the breakpoint cluster region (BCR)-Abl fusion protein, which occurs frequently in chronic myelogenous leukaemia (CML) as a result of a chromosomal translocation event [22–28]. While small-molecule tyrosine kinase inhibitors (TKIs) of BCR-Abl, such as Imatinib, have had great success in the clinic, acquired drug resistance remains a problem in treating CML effectively [29]. As such, SHP2 has been considered a useful therapeutic target and the monobodies recognizing SHP2 (used in this study) were reported to inhibit MAPK signalling driven by BCR-Abl overexpression [17]. More recently, an allosteric inhibitor of SHP2, SHP099, was also reported to inhibit proliferation and MAPK signalling in cancers driven by receptor tyrosine kinases (RTKs) [21].

ASC is a key common adaptor of all inflammasomes, which are key signalling platforms in myeloid cells that detect infections and cell damage. Inflammasomes enable the production of pro-inflammatory cytokines IL-1β and IL-18, as well as cell death by pyroptosis [30,31]. ASC knockout mice exhibit reduced levels of pro-inflammatory cytokines in the serum, while bone marrow-derived macrophages from these mice display defective inflammasome signalling [32]. Overexpression of the nanobody recognizing human ASC has been shown to impair inflammasome assembly and signalling [16].

Equipped with small polypeptide monobodies recognizing SHP2 and a nanobody recognizing human ASC protein, we sought to test whether the proteolytic AdPROM system, consisting of VHL conjugated to these mono/nanobodies, was capable of degrading endogenous SHP2 and ASC via the proteasome across different cell lines. In this study, we show for the first time that the proteolytic AdPROM system can be engineered with selective mono/nanobodies recognizing endogenous target proteins to efficiently degrade the target protein in many cell types.

## 2. Results

### 2.1. Proteolytic AdPROM degrades endogenous SHP2 in multiple human cancer cell lines

**Figure 1.**
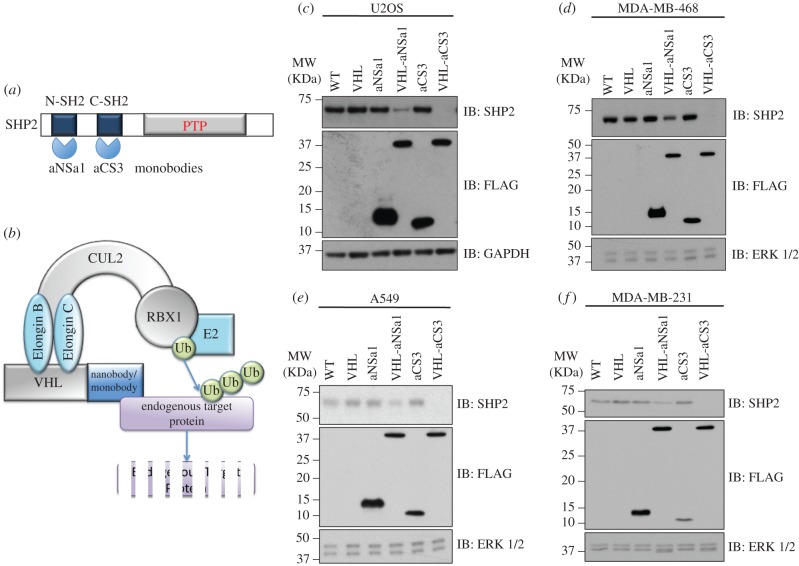
Proteolytic AdPROM degrades endogenous SHP2 in multiple human cancer cells. (*a*) Domain structure of SHP2. N-SH2, N-terminal SH2 domain; C-SH2, C-terminal SH2 domain; PTP, protein tyrosine phosphatase. The monobodies aNSa1 and aCS3 recognize the N-SH2 and C-SH2 domains, respectively. (*b*) Schematic describing the concept of AdPROM-mediated target protein degradation using monobodies or nanobodies to recruit endogenous target proteins. For example, VHL conjugated to SHP2 monobody recruits endogenous SHP2 protein to the EloB/C/CUL2/RBX1 E3 ligase machinery for ubiquitin-mediated proteasomal degradation. (*c–f*) Human U2OS osteosarcoma (*c*), MDA-MB-468 mammary gland adenocarcinoma (*d*), A549 lung cancer (*e*) and MDA-MB-231 breast cancer (*f*) cells were infected with retroviruses encoding FLAG-VHL-aNSa1 or FLAG-VHL-aCS3 or controls (VHL alone, FLAG-aNSa1 alone and FLAG-aCS3 alone) as indicated or left uninfected (WT). Cell extracts (20 µg protein) were resolved by SDS-PAGE and transferred to PVDF membranes, which were subjected to western blotting with the indicated antibodies.

In an attempt to test whether the proteolytic AdPROM consisting of VHL tethered to monobodies recognizing human SHP2 could degrade endogenous SHP2, we employed two distinct monobodies that selectively bind the N-SH2 and C-SH2 domains of SHP2, termed aNSa1 (*K*_D_ = 14 nM) and aCS3 (*K*_D_ = 4 nM), respectively [17] (figure 1*a,b*). VHL-aNSa1 and VHL-aCS3 were packaged in mammalian expression pBABE retroviral vectors, with an in-frame FLAG tag at the N-terminus for monitoring AdPROM expression. We infected U2OS osteosarcoma, MDA-MB-468 mammary gland adenocarcinoma, A549 pulmonary adenocarcinoma and MDA-MB-231 breast cancer cells with retroviruses encoding the expression of VHL, aNSa1, VHL-aNSa1, aCS3 or VHL-aCS3, and monitored the endogenous SHP2 levels (figure 1*c–f*). The endogenous SHP2 levels were comparable and unaltered in uninfected cells and cells infected with VHL, aNSa1, or aCS3 controls (figure 1*c–f*). By contrast, compared with these control cells, we observed a substantial reduction in endogenous SHP2 protein levels upon expression of VHL-aNSa1 (figure 1*c–f*). Strikingly, no detectable SHP2 protein levels were observed in any of the cell lines when VHL-aCS3 was expressed in cells (figure 1*c–f*). This reduction in SHP2 protein levels is unlikely to be due to a decrease in SHP2 mRNA transcripts, as cells infected with VHL-aCS3 appeared to express slightly higher levels of SHP2 transcripts compared with the control cells (electronic supplementary material, figure S1). Collectively, the results suggest that the VHL-aCS3 AdPROM results in complete proteolysis of endogenous SHP2 in multiple human cancer cell lines.

### 2.2. Proteolytic AdPROM uses CUL2-CRL for SHP2 destruction

**Figure 2.**
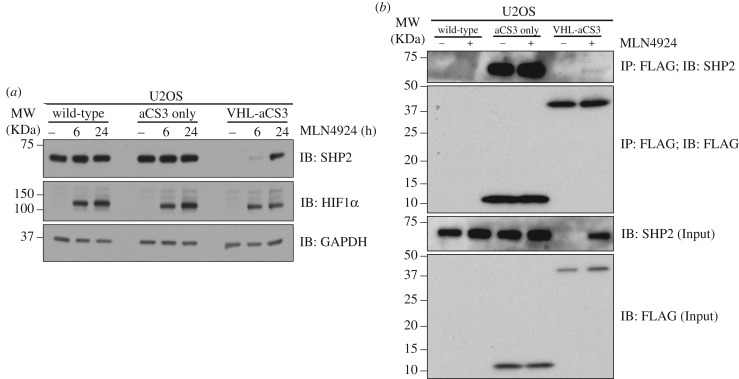
Proteolytic AdPROM employs CUL2-CRL for SHP2 destruction. (*a*) Uninfected U2OS cells (WT) or cells infected with retroviruses encoding FLAG-VHL-aCS3 or control viruses (FLAG-aCS3 alone) were treated with MLN4924 (1 µM) for 0, 6 or 24 h. Cells were lysed and extracts (20 µg protein) were resolved by SDS-PAGE and transferred to PVDF membranes, which were subjected to western blotting with the indicated antibodies. HIF1α serves as a positive control for MLN4924 treatment. (*b*) U2OS cells treated with or without MLN4924 (1 µM) for 24 h were lysed and extracts (1 mg protein) subjected to immunoprecipitation (IP) with anti-FLAG M2 resins. Following elution, both IPs and input extracts (20 µg protein) were resolved by SDS-PAGE and transferred to PVDF membranes, which were subjected to western blotting with the indicated antibodies.

To demonstrate that degradation of SHP2 by VHL-aNSa1 and VHL-aCS3 occurs via the predicted CUL2-CRL machinery, we used the selective Cullin NEDDylation inhibitor MLN4924 in U2OS cells. In uninfected wild-type U2OS cells or control cells infected with aCS3, we observed no notable changes in levels of endogenous SHP2 protein following treatment of cells with MLN4924 for 6 or 24 h (figure 2*a*). By contrast, the degradation of endogenous SHP2 caused by VHL-aCS3 was rescued in a time-dependent manner following MLN4924 treatment, with a robust rescue observed at 24 h (figure 2*a*). Analogous rescue of SHP2 protein levels by MLN4924 treatment was observed in U2OS cells infected with VHL-aNSa1 AdPROM (electronic supplementary material, figure S2*a*). Consistent with the key role of the CUL2-CRL machinery in controlling hypoxia-inducible factor 1-alpha (HIF1α) protein stability, treatment of cells with MLN4924 resulted in stabilization of HIF1α at both 6 h and 24 h time points (figure 2*a*; electronic supplementary material, figure S2a). Infection of cells with any of aCS3, VHL-aCS3, aNSa1 and VHL-aNSa1 did not result in changes in HIF1α protein levels (figure 2*a*; electronic supplementary material, figure S2a), suggesting that the expression of the AdPROM system does not interfere with hypoxia signalling.

Next, we sought to verify the interaction between the monobodies and the endogenous SHP2 in cell extracts. We performed anti-FLAG immunoprecipitations (IPs) from extracts of wild-type U2OS cells or those infected with FLAG-aCS3 or FLAG-VHL-aCS3 and treated with or without MLN4924 (figure 2*b*). FLAG-aCS3 IPs robustly precipitated endogenous SHP2, irrespective of MLN4924 treatment, while control anti-FLAG IPs from wild-type U2OS extracts did not pull down SHP2 (figure 2*b*). By contrast, in the absence of MLN4924 treatment, FLAG-VHL-aCS3 IPs did not precipitate detectable levels of endogenous SHP2, while following MLN4924 treatment some endogenous SHP2 was detected (figure 2*b*). These observations simply reflect the levels of endogenous SHP2 detected in extracts of cells infected with FLAG-VHL-aCS3 (figure 2). Analogous results were observed when cells were infected with FLAG-aNSa1 and FLAG-VHL-aNSa1 (electronic supplementary material, figure S2*b*). Collectively, these results highlight the affinity of aNSa1 and aCS3 for endogenous SHP2 and suggest that the proteolytic AdPROM-mediated degradation of SHP2 is mediated by the VHL-CUL2 machinery.

### 2.3. SHP2 destruction causes partial attenuation of ERK1/2 phosphorylation

**Figure 3.**
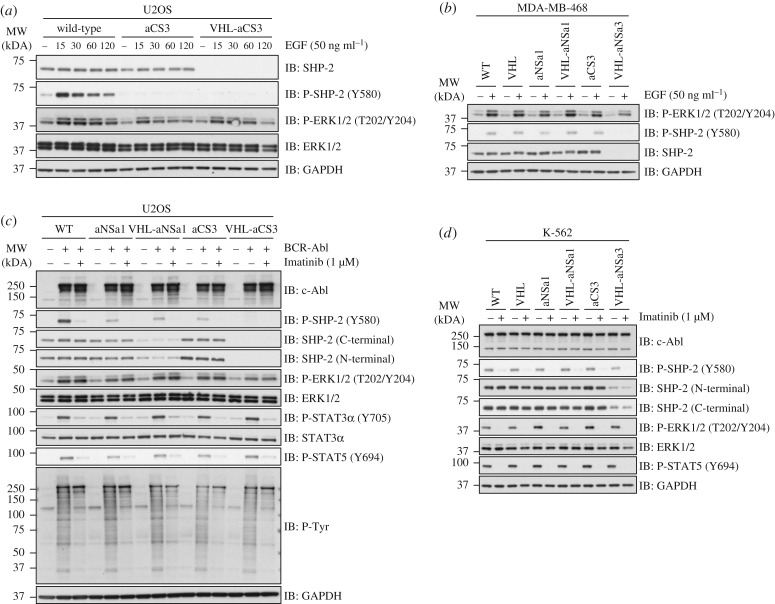
SHP2 destruction causes partial attenuation of ERK1/2 phosphorylation. (*a*) Wild-type, uninfected U2OS cells or cells infected with FLAG-aCS3 and FLAG-VHL-aCS3 were treated with EGF (50 ng ml^−1^) and lysed at indicated time points. Cell extracts (10 µg protein) were resolved by SDS-PAGE and transferred to nitrocellulose membranes, which were subjected to western blotting with the indicated antibodies. (*b*) As in (*a*), except that uninfected wild-type (WT) MDA-MB-468 cells or cells infected with retroviruses encoding VHL, FLAG-aNSa1, FLAG-VHL-aNSa1, FLAG-aCS3 and FLAG-VHL-aCS3 were left untreated or treated with EGF for 30 min prior to lysis. (*c*) Uninfected (WT) U2OS cells or cells infected with retroviruses encoding VHL, FLAG-aNSa1, FLAG-VHL-aNSa1, FLAG-aCS3 and FLAG-VHL-aCS3 were transfected with HA-BCR-Abl fusion gene. Cells were treated with or without the BCR-Abl kinase inhibitor Imatinib (1 µM) for 4 h prior to lysis. Cells were lysed and extracts (10 µg protein) were resolved by SDS-PAGE and transferred to nitrocellulose membranes, which were subjected to western blotting with the indicated antibodies. (*d*) Human erythroleukaemia K-562 cells harbouring the natural BCR-Abl transgene mutation were infected with retroviruses encoding VHL, FLAG-aNSa1, FLAG-VHL-aNSa1, FLAG-aCS3 and FLAG-VHL-aCS3 or left uninfected (WT). Cells were treated with or without Imatinib (1 µM) for 2 h prior to lysis. Cells were lysed and extracts (10 µg protein) were resolved by SDS-PAGE and transferred to nitrocellulose membranes, which were subjected to western blotting with the indicated antibodies.

SHP2 activity has been reported to regulate Ras/MAPK signalling [17,33]. In order to evaluate the impact of AdPROM-mediated proteolysis of SHP2 in cells, we assessed the levels of basal and epidermal growth factor (EGF)-induced phosphorylation of ERK1/2 (also referred to as p44/p42 MAPK) in U2OS and MDA-MB-468 cells. First, we performed a time course experiment for EGF stimulation in wild-type U2OS cells or cells infected with either aCS3 or VHL-aCS3. In wild-type cells, EGF induced a peak ERK1/2 phosphorylation at Thr^202^ and Tyr^204^ residues between 15 and 60 min, which diminished after 120 min of EGF treatment (figure 3*a*). Expression of aCS3 alone, which binds the C-SH2 domain of SHP2 and inhibits it allosterically [17] and does not cause SHP2 degradation, caused partial reduction in levels of EGF-induced phospho-ERK1/2 at all time points tested (figure 3*a*). Similarly, VHL-aCS3 expression, which caused complete loss of SHP2 protein, also caused partial reduction in levels of EGF-induced phospho-ERK1/2 at all time points tested (figure 3*a*). Both aCS3 and VHL-aCS3 expression caused complete loss of SHP2 phosphorylation at Tyr^580^ (figure 3*a*). These results suggest that both occluding the C-SH2 domain of SHP2 by aCS3 monobody and the loss of SHP2 protein both equally impact EGF-induced phosphorylation of ERK1/2 in U2OS cells. By contrast, in MDA-MB-468 cells in which SHP2 has been reported to mediate Ras/MAPK signalling downstream of receptor tyrosine kinases, only destruction of SHP2 by VHL-aCS3 caused a moderate reduction in EGF-induced phospho-ERK1/2 levels, while the expression of VHL, aNSa1, VHL-aNSa1 or aCS3 alone did not significantly impact EGF-induced phospho-ERK1/2 levels or SHP2 phosphorylation at Tyr^580^ (figure 3*b*).

Activation of Ras/MAPK signalling downstream of the constitutively active BCR-Abl tyrosine kinase has been reported to require SHP2 activity [17]. To investigate the effects of AdPROM-mediated SHP2 degradation in the context of BCR-Abl-induced Ras/MAPK signalling, we transfected the BCR-Abl fusion gene into wild-type U2OS cells or cells infected with aNSa1, VHL-aNSa1, aCS3 or VHL-aCS3, and monitored levels of phospho-ERK1/2. In wild-type control cells, BCR-Abl expression resulted in robust SHP2 phosphorylation on Tyr^580^, a known c-Abl phosphorylation site on SHP2 [34], and phosphorylation of ERK1/2 (figure 3*c*). Treatment of cells with Imatinib, the BCR-Abl tyrosine kinase inhibitor, completely abolished phosphorylation of SHP2 on Tyr^580^, STAT3 on Tyr^705^ and STAT5 on Tyr^694^ [31–34], but did not significantly impact BCR-Abl-induced phospho-ERK1/2 levels (figure 3*c*). In cells infected with aNSa1, VHL-aNSa1 and aCS3, the BCR-Abl-induced phosphorylation of ERK1/2 was not significantly different from wild-type U2OS cells (figure 3*c*). However, in cells infected with VHL-aCS3, which caused complete degradation of SHP2, BCR-Abl-induced phospho-ERK1/2 levels were slightly attenuated compared with wild-type and aCS3 alone controls (figure 3*c*). As reported previously [17], aNSa1 and aCS3 expression caused reduction in SHP2 phosphorylation on Tyr^580^, suggesting that the interactions between these monobodies and SHP2 potentially obstruct the BCR-Abl-induced phosphorylation of SHP2 (figure 3*c*). The phosphorylation of SHP2 was undetectable in cells infected with VHL-aCS3, as there was no detectable level of the total SHP2 protein (figure 3*c*). The infection of cells with aNSa1, VHL-aNSa1, aCS3 and VHL-aCS3 did not alter the levels of phospho-STAT3-Tyr^705^ or phospho-STAT5-Tyr^694^ (figure 3*c*). Compared with WT cells or cells infected with VHL, aNSa1 and VHL-aNSa1, infection of cells with aCS3 and VHL-aCS3 caused a reduction in BCR-Abl-induced total phospho-Tyr levels (figure 3*c*). Similarly, Imatinib treatment also caused reduction in BCR-Abl-induced total phospho-Tyr levels (figure 3*c*).

We also tested the effects of AdPROM-mediated SHP2 degradation on MAPK signalling in K-562 chronic myelogenous leukaemia cells, which naturally harbour the BCR-Abl fusion (figure 3*d*). We infected K-562 cells with retroviruses encoding the expression of VHL, aNSa1, VHL-aNSa1, aCS3 or VHL-aCS3, treated them with or without the BCR-Abl tyrosine kinase inhibitor Imatinib, and monitored the SHP2 and phospho-ERK1/2 levels (figure 3*d*). The endogenous SHP2 levels were comparable and unaltered in uninfected cells or cells infected with VHL, aNSa1 or aCS3 controls. By contrast, compared with these control cells, we observed a slight reduction in endogenous SHP2 protein levels upon expression of VHL-aNSa1, but a substantial reduction upon VHL-aCS3 expression (figure 3*d*). Under these conditions, in the absence of Imatinib the levels of phospho-ERK1/2 did not change substantially (figure 3*d*). By contrast, treatment of cells with Imatinib caused complete inhibition of phospho-ERK1/2 and phospho-STAT5 levels, both of which are known to be induced by BCR-Abl [17,22,24,25,28] (figure 3*d*). Collectively, these observations suggest that the role of SHP2 in Ras/MAPK signalling is perhaps limited in nature and may be context-specific.

### 2.4. Comparing the effects of SHP2 degradation and allosteric inhibition on Ras/MAPK signalling

**Figure 4.**
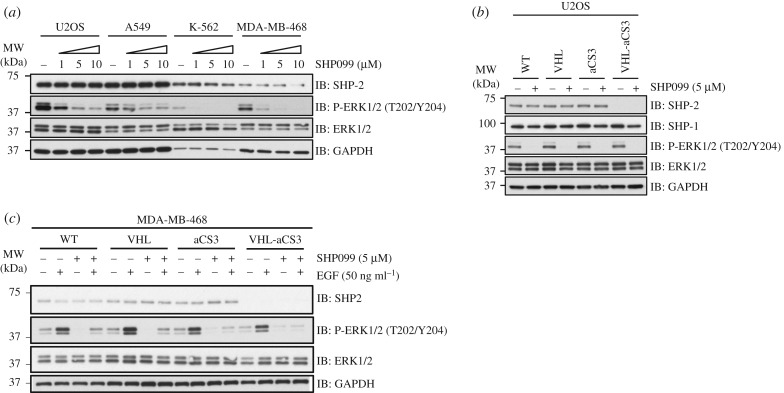
Comparing the effects of SHP2 degradation and allosteric inhibition on Ras/MAPK signalling. (*a*) Human U2OS, A549, K-562 and MDA-MB-468 cells were treated with DMSO control or 1, 5 and 10 µM SHP099 for 2 h prior to lysis. Extracts (10 µg protein) were resolved by SDS-PAGE and transferred to nitrocellulose membranes, which were subjected to western blotting with the indicated antibodies. (*b*) Uninfected U2OS cells (WT) or cells infected with retroviruses encoding VHL, aCS3 and VHL-aCS3 were treated with or without 5 µM SHP099 for 2 h prior to lysis. Cell extracts (10 µg protein) were resolved by SDS-PAGE and transferred to nitrocellulose membranes, which were subjected to western blotting with the indicated antibodies. (*c*) Uninfected MDA-MB-468 cells (WT) or cells infected with retroviruses encoding VHL, aCS3 or VHL-aCS3 were treated with or without 5 µM SHP099 for 2 h, and stimulated with or without EGF (50 ng ml^−1^) for the last 30 min prior to lysis. Cell extracts (20 µg protein) were processed as described in (*b*).

The context-dependent effects of SHP2 in Ras/MAPK signalling were illustrated in a recent report in which a selective allosteric inhibitor of SHP2, termed SHP099, was shown to block the activation of ERK1/2 downstream of RTKs in certain cell types [21]. We treated a number of cell lines with varying doses of SHP099 to assess the impact of SHP2 inhibition on ERK1/2 phosphorylation (figure 4*a*). We observed that SHP099 caused an efficient, dose-dependent inhibition of ERK1/2 phosphorylation in U2OS, K-562 and MDA-MB-468 cells, but not an efficient inhibition in A549 cells (figure 4*a*). It should be noted that the relative levels of SHP2 in U2OS and A549 cells were higher than K-562 and MDA-MB-468 cells when normalized against the total protein concentration in extracts (figure 4*a*). In order to investigate whether the inhibition of ERK1/2 phosphorylation by SHP099 is mediated through SHP2, we tested the impact of AdPROM-mediated degradation of SHP2 in U2OS cells treated with or without SHP099 (figure 4*b*). In wild-type U2OS cells, basal phospho-ERK1/2 levels were completely inhibited by SHP099 (figure 4*b*). Compared with the wild-type cells, no significant changes in the levels of phospho-ERK1/2 were observed in U2OS cells infected with VHL, aCS3 or VHL-aCS3, which caused complete degradation of endogenous SHP2 but not SHP1 (figure 4*b*). In cells infected with VHL-aCS3, SHP099 still resulted in complete loss in phospho-ERK1/2 levels (figure 4*b*), suggesting that the effect of SHP099 on MAPK signalling in these cells is mediated, at least in part, through an off-target mechanism. Interestingly, SHP099 treatment appeared to cause a slight reduction in levels of endogenous SHP1 protein (figure 4*b*).

To mitigate any possibility that the effects of SHP099 on MAPK signalling in SHP2-depleted U2OS cells were context-specific, we used the MDA-MB-468 cells that were employed to demonstrate the allosteric inhibition of SHP2 by SHP099 [21]. In order to explore the effects of SHP099 on MAPK signalling in these cells, we treated wild-type MDA-MB-468 cells or cells infected with VHL, aCS3 alone or VHL-aCS3 AdPROM retroviruses, with EGF in the presence or the absence of SHP099. In wild-type MDA-MB-468 cells, EGF induced a robust ERK1/2 phosphorylation, while SHP099 completely blocked this phosphorylation (figure 4*c*). We observed the same effects in cells infected with either VHL or aCS3 alone retroviruses, which did not alter the levels of SHP2 protein compared with the wild-type controls (figure 4*c*). In MDA-MB-468 cells infected with VHL-aCS3, in which no detectable SHP2 protein levels were observed, EGF still induced robust ERK1/2 phosphorylation, albeit slightly lower compared with wild-type control, and SHP099 treatment still resulted in complete inhibition of EGF-induced ERK1/2 phosphorylation (figure 4*c*). Given the striking reduction in phospho-ERK1/2 levels caused by SHP099 under conditions where SHP2 protein levels are undetectable, the effects of SHP099 are likely to be mediated, at least in part, independently of SHP2 inhibition.

### 2.5. Comparing proteolytic AdPROM with siRNA for the depletion of SHP2 protein

**Figure 5.**
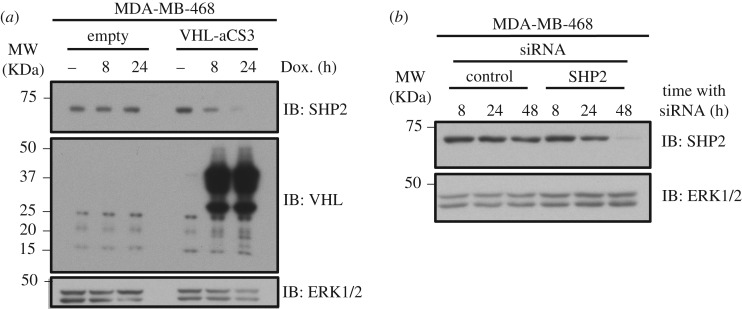
Comparing proteolytic AdPROM with siRNA for the depletion of SHP2 protein. (*a*) MDA-MB-468 cells were infected with retroviruses encoding pRetroX-Tet-On transactivator and either pRetroX-Tight empty vector or pRetroX-Tight-VHL-aCS3. Infected cells were treated with doxycycline (2 µg ml^−1^) for the indicated time points prior to lysis. Cell extracts (20 µg protein) were subjected to SDS-PAGE and transferred to PVDF membranes, which were subjected to western blotting with the indicated antibodies. (*b*) MDA-MB-468 cells were transfected with non-targeting control siRNAs or siRNAs targeting SHP2 and lysed at the indicated time points after transfection. Cell extracts (20 µg protein) were subjected to SDS-PAGE and transferred to PVDF membranes, which were subjected to western blotting with the indicated antibodies.

In order to evaluate the rate of proteolysis caused by VHL-aCS3 AdPROM, we packaged VHL-aCS3 into a *Tet*-inducible pRetroX-Tight plasmid and introduced it to MDA-MB-468 cells, along with the pRetroX-Tet-On transactivator plasmid. Treatment of these cells, but not control cells, with doxycycline induced a time-dependent increase in expression of VHL-aCS3 and a concurrent reduction in SHP2 protein levels (figure 5*a*). In cells infected with VHL-aCS3, SHP2 protein levels were substantially reduced at 8 h and almost completely depleted at 24 h after doxycycline treatment (figure 5*a*), suggesting that the expression of VHL-aCS3 induces a rapid degradation of SHP2 protein.

In order to test how AdPROM-mediated SHP2 degradation compared with siRNA-mediated depletion, we transfected MDA-MB-468 cells with either control siRNAs or siRNA pool targeting SHP2 and lysed cells at 8, 24 and 48 h after transfection. As expected, the control siRNAs did not affect the levels of SHP2 protein at all times tested (figure 5*b*). By contrast, siRNAs against SHP2 did not deplete SHP2 protein levels at 8 and 24 h after transfection but caused substantial reduction in SHP2 levels only 48 h after transfection (figure 5*b*). These results clearly demonstrate that siRNA-mediated loss in target proteins require longer treatments compared with the proteolytic AdPROM.

### 2.6. Proteolytic AdPROM is adaptable for degradation of endogenous ASC

**Figure 6.**
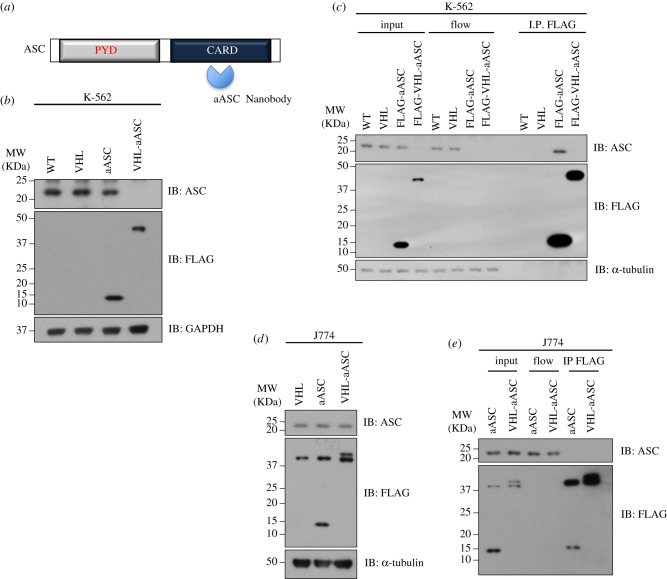
Proteolytic AdPROM is adaptable for degradation of endogenous ASC. (*a*) Domain structure of ASC with the N-terminal Pyrin domain (PYD) and C-terminal Caspase recruitment domain (CARD) indicated. The aASC nanobody recognizes the CARD domain of human ASC. (*b*) Uninfected human K-562 cells (WT) or cells infected with retroviruses encoding VHL, FLAG-aASC or FLAG-VHL-aASC were lysed. Extracts (20 µg protein) were resolved by SDS-PAGE and transferred to PVDF membranes, which were subjected to western blotting with the indicated antibodies. (*c*) Extracts (1 mg protein) from (*b*) were subjected to IP with anti-FLAG M2 resins. The resulting IPs and input and flow-through extracts (20 µg protein) were resolved by SDS-PAGE and transferred to PVDF membranes, which were subjected to western blotting with the indicated antibodies. (*d*) As in *(b*) except that murine J774 monocyte cells were used. (*e*) As in (*c*) except that murine J774 monocytes from (*d*) were used.

The efficacy of AdPROM-mediated destruction of endogenous SHP2 from multiple cell lines prompted us to deploy the system to explore the efficiency of degradation of other distinct endogenous proteins, for which selective and potent nanobodies have been reported. We adapted the proteolytic AdPROM system by incorporating a nanobody recognizing the human ASC protein (aASC; figure 6*a*), in the same way as we used aNSa1 and aCS3 for SHP2 destruction. VHL-aASC was packaged into mammalian expression pBABE retroviral vector, with a FLAG-tag at the N-terminus for monitoring AdPROM expression. We infected ASC-expressing K-562 cells with retroviruses encoding the expression of VHL, aASC or VHL-aASC and monitored the endogenous ASC levels (figure 6*b*). The endogenous ASC levels remained comparable and unaltered in uninfected cells and cells infected with VHL and aASC controls. Strikingly, no detectable endogenous ASC protein levels were observed in K-562 cells infected with VHL-aASC, indicating a robust proteolytic AdPROM system for complete destruction of endogenous human ASC protein (figure 6*b*). In order to demonstrate the affinity of the aASC nanobody for human ASC protein, anti-FLAG IPs from FLAG-aASC infected cell extracts robustly precipitated endogenous ASC and depleted it from the flow-through extracts, while control IPs from uninfected or FLAG-VHL-infected cell extracts did not (figure 6*c*). As ASC was completely degraded by VHL-aASC, IPs of VHL-aASC did not precipitate detectable levels of ASC protein (figure 6*c*). Consistent with the affinity of aASC nanobody only to human (and not mouse) ASC protein, the VHL-aASC AdPROM, despite robust expression, did not degrade endogenous ASC protein in murine J774 monocytes (figure 6*d*). Consistent with this, IPs of aASC and VHL-aASC did not precipitate endogenous ASC protein from these murine cells (figure 6*e*). These data suggest that AdPROM-mediated destruction of human (but not mouse) ASC protein relies on the affinity of the nanobody to the human protein. Collectively, our data provide solid evidence that nanobodies recognizing endogenous target proteins can be deployed using the proteolytic AdPROM system to achieve robust degradation of endogenous protein targets in any cell line.

## 3. Discussion

Efficient targeted proteolysis of endogenous proteins in cells can serve as a powerful tool to inhibit protein function, both as a research tool and in therapeutics. In this report, we describe a robust proteolytic AdPROM system to achieve effective and efficient destruction of endogenous target proteins, both constitutively and in an inducible manner. By using high-affinity polypeptide monobody binders of human SHP2 and a nanobody recognizing human ASC protein conjugated to the CUL2 substrate receptor VHL, we achieve near-complete proteolysis of endogenous SHP2 and ASC in human cancer cell lines. We provide evidence that the AdPROM-mediated proteolysis of endogenous SHP2 requires the CRL-dependent UPS and can be rescued by the Cullin NEDDylation inhibitor MLN4924. Furthermore, the expression of the proteolytic AdPROM in cells does not appear to impact the stability of the *bona fide* VHL-target HIF1α.

Gene knockouts are clearly irreversible and for many genes they are not feasible. Similarly, as we have shown, RNA interference approaches require long treatments to permit protein depletion and are also associated with off-target effects. Therefore, rapid and direct destruction of target proteins by the proteolytic AdPROM system and proteolysis targeting chimeras (PROTACs) technology, which can overcome these limitations [12,35], are desirable. We have demonstrated that proteolytic AdPROM can be applied efficiently for both inducible and constitutive degradation of target proteins [12]. While the development of PROTACs against protein targets can be extremely tedious and expensive, the proteolytic AdPROM system can be assembled rapidly and its flexibility makes it easily adaptable for any number of E3 ubiquitin ligases. The recent advances in synthetic and antibody-derived monobody and nanobody technology platforms [13,14] mean that the portfolio of both monobodies and nanobodies targeting individual proteins of interest is certain to see an exponential rise in the near future, thus increasing the target pool for the proteolytic AdPROM system. Even in the absence of individual protein-targeting monobodies and nanobodies, we have shown how the proteolytic AdPROM system can be applied relatively quickly, by first generating a GFP knock-in (KI) at the target loci with CRISPR/Cas9 [12]. The proteolytic AdPROM system can rapidly inform the potential effectiveness of PROTACs as an approach against specific targets.

By using the VHL-aCS3 AdPROM, we were able to completely degrade endogenous SHP2 from U2OS, A549, MDA-MB-231 and MDA-MB-468 cells, and robustly deplete SHP2 levels from K-562 cells. When compared with control cells, constitutive SHP2 degradation did not substantially alter (A549 and K-562) or only moderately attenuated (U2OS, MDA-MB-468) the levels of pERK1/2. These observations are consistent with many reports that imply contrasting context-specific roles of SHP2 in Ras/MAPK signalling [17,19–21,36]. Recently, a small-molecule allosteric SHP2 inhibitor, SHP099, was reported to inhibit RTK-dependent Ras/MAPK signalling and cancer cell proliferation in a number of cell lines, including MDA-MB-468 mammary gland adenocarcinoma cells [21]. In this study, we too showed that SHP099 potently inhibited pERK1/2 in MDA-MB-468 cells, as well as U2OS, A549 and K562 cells. However, the AdPROM-mediated destruction of SHP2 in MDA-MB-468 and U2OS cells did not inhibit (or only partially inhibited) pERK1/2 levels. These observations imply that either the AdPROM-mediated SHP2 depletion, despite resulting in undetectable levels, is still insufficient to completely block Ras/MAPK signalling or the effect of SHP099 on Ras/MAPK signalling is mediated, at least in part, through off-target effects. The SHP2-dependent inhibition of SHP099 on ERK1/2 phosphorylation was in part bolstered by overexpressing the SHP099-interaction-deficient mutant of SHP2 (T253M/Q257L) in KYSE520 cells, which restored the pERK1/2 levels [21]. However, in the light of our data, and with many other studies reporting contradictory roles of SHP2 on Ras/MAPK signalling as described above, more work is required to establish the precise, context-dependent mechanisms of SHP2 action in Ras/MAPK signalling. The simplicity and broad applicability of the AdPROM system for complete destruction of SHP2 in many human cell lines means that it can facilitate rapid investigations into SHP2 function and substrate identification.

Using the proteolytic AdPROM with aASC nanobody that selectively recognizes the human ASC protein, we also achieved complete destruction of the ASC protein in human K-562 cells but not in murine J774 cells. These observations clearly illustrate the affinity-driven nature of the AdPROM system. The functional tests on the loss of ASC protein from K-562 cells proved challenging, as these cells were unresponsive to signals that activate the inflammasome signalling pathway. Nonetheless, AdPROM-mediated destruction of ASC will be useful for investigating inflammasome signalling in relevant contexts, such as monocytes. Consistent with the notion that affinity is key to proteolysis of the target proteins, if AdPROM is used with monobodies and nanobodies that selectively recognize a particular state of the target protein (e.g. post-translational modification, active or inactive state, or mutation), then it would be possible, in principle, to degrade that pool of protein, allowing functional targeting.

With increasing pharmaceutical interest in PROTACs [37,38], the AdPROM-mediated recruitment and subsequent ubiquitination of specific target proteins presents a novel and tantalizing therapeutic opportunity. While PROTACs are expensive and time-consuming to develop, AdPROM is quick and robust, and can be exploited as a research tool to explore the suitability of a protein target as a CRL substrate, prior to investing time and resources into PROTAC development. If and when gene delivery technologies become efficient and routine, AdPROM could be used effectively in therapeutics.

## 4. Material and methods

### 4.1. Plasmids and reagents

The cDNAs encoding FLAG-aNSa1 (DU54816), FLAG-VHL-aNSa1 (DU54843), FLAG-aCS3 (DU54817), FLAG-VHL-aCS3 (DU54844), FLAG-aASC (DU54821), FLAG-VHL-aASC (DU54832) and human VHL (DU54023) were designed from published protein sequences [16,17] and cloned into pBABED-Puro vectors (Cell Biolabs, modified; Dundee-modified version of the original Cell Biolabs pBABE plasmid) for constitutive expression. The retroviral expression system vectors pCMV-Gag-Pol and pCMV-VSVG constructs were from Clontech. pCDNA5 FRT/TO-HA-BCR-Abl (DU26928) was transiently expressed in cells where indicated in the figure legends. All DNA constructs were verified by DNA sequencing, performed by the DNA Sequencing and Services (MRCPPU, College of Life Sciences, University of Dundee, Scotland, http://www.dnaseq.co.uk) using Applied Biosystems Big-Dye v. 3.1 chemistry on an Applied Biosystems model 3730 automated capillary DNA sequencer. All constructs are available to request from the MRC-PPU reagents webpage (http://mrcppureagents.dundee.ac.uk) and the unique identifier (DU) numbers indicated above provide direct links to the cloning and sequence details. The pRetroX-Tet-On and pRetroX-Tight vectors for inducible AdPROM expression were obtained from Clontech. The non-targeting control siRNAs (cat.: D-001810-01-05) and the SHP2 (PTPN11) siRNA pool (cat.: L-003947-00-0005) were from Dharmacon.

### 4.2. Antibodies

Rabbit anti-GAPDH (cat.: 2118), anti-SHP2 (C-term) (cat.: D50F2), anti-SHP2 (N-term) (cat.: 3752), anti-pSHP2 Tyr^580^ (cat.: 5431), anti-pERK1/2(Thr^202^/Tyr^204^) (cat.: 9101), anti-ERK1/2 (cat.: 4695), anti-pSTAT3 (Tyr^705^)(cat.: 9131), anti-STAT3 (cat.: 8768), anti-VHL (cat.: 68547), anti-pSTAT5 (Tyr^694^) (cat.: 9359) and anti-c-Abl (cat.: 2862) antibodies were purchased from CST. Anti-HIF1α antibody (cat.: 610959) was purchased from BD Transduction Laboratories. Anti-FLAG M2-peroxidase (HRP) (cat.: A8592) was purchased from Sigma. Anti-phospho-tyrosine (cat.: 05321) antibody was purchased from Millipore. Anti-ASC antibody (cat.: AL177) was purchased from Adipogen. For western blot analysis, all primary antibodies were used at 1 : 1000 dilution, except for anti-GAPDH antibody, and anti-FLAG M2-peroxidase (HRP), which were used at 1 : 5000 and 1 : 2000 dilutions, respectively. Horseradish peroxidase (HRP)-coupled secondary antibodies (1 : 5000) were obtained from Santa Cruz. Anti-FLAG M2 resin for immunoprecipitation was from Sigma (cat.: A2220).

### 4.3. Cell culture and lysis

Human U2OS osteosarcoma, A549 pulmonary adenocarcinoma cells and mouse J774 monocyte were cultured in Dulbecco's Modified Eagle's Medium (DMEM; Gibco) supplemented with 10% (v/v) fetal bovine serum (FBS) (Hyclone), 2 mM l-glutamine (Lonza), and 1% (v/v) penicillin/streptomycin (Lonza) and maintained at 37°C in a humidified incubator at 5% CO_2_. Human MDA-MB-468 mammary gland adenocarcinoma, MDA-MB-231 breast cancer and K-562 erythroleukaemia cells were cultured in RPMI medium (Gibco) supplemented with 10% (v/v) fetal bovine serum (FBS) (Hyclone), 2 mM l-glutamine (Lonza) and 1% (v/v) penicillin/streptomycin (Lonza), and maintained at 37°C in a humidified incubator at 5% CO_2_. Cells were treated with varying compounds and stimuli as described in the appropriate figure legends. For retroviral production, pBABED retroviral plasmids (6 µg) encoding appropriate proteins were co-transfected with pCMV-gag-pol (3.2 µg) and pCMV-VSV-G (2.8 µg) in a 10 cm–diameter dish of 70% confluent 293-FT cells. In short, plasmids were mixed in 600 µl Opti-MEM Reduced Serum Medium (Life Technologies) to which 24 µl of 1 mg ml^−1^ polyethylenimine (PEI) (Polysciences Inc.) diluted in 25 mM HEPES pH 7.5 was added. Following 20 s of vortexing and incubation for 20 min at room temperature, the resulting transfection solution was applied drop-wise to the 293-FT cells. The medium was replaced 16 h post-transfection with fresh full medium, followed by collection of the retroviruses in the growth medium 24 h later, which were filtered using 0.45 µm filters. Target cells (approx. 60% confluent) were infected with the optimized titre of retroviral medium containing 8 µg ml^−1^ polybrene (Sigma) for 24 h, as described previously [12,39,40]. Cells which had integrated the retrovirus were selected with 2 µg ml^−1^ puromycin thereafter.

For lysis, cells were washed twice in ice-cold phosphate-buffered saline (PBS), scraped on ice in lysis buffer (50 mM Tris–HCl pH 7.5, 0.27 M sucrose, 150 mM NaCl, 1 mM EGTA, 1 mM EDTA, 1 mM sodium orthovanadate, 10 mM sodium β-glycerophosphate, 50 mM sodium fluoride, 5 mM sodium pyrophosphate, 1% (v/v) Nonidet P-40) supplemented with complete protease inhibitors (one tablet per 25 ml; Roche) and 0.1% (v/v) β-mercaptoethanol (Sigma). Cell extracts were clarified and either processed immediately or snap-frozen in liquid nitrogen prior to storage at −80°C. The protein concentration was determined in a 96-well format using Bradford protein assay reagent (Pierce). For anti-FLAG M2 immunoprecipitation, lysates (typically 1 mg) were incubated with anti-FLAG M2 resin (10 µl) for 16 h overnight at 4°C on a rotating wheel. Following washing of the resin with lysis buffer 3×, FLAG-fusion proteins were eluted from the resin by boiling for 5 min at 95°C in 1× SDS sample buffer. Samples were then processed for SDS-PAGE as described above.

### 4.4. Growth factors and small-molecule inhibitors

Purified recombinant human epidermal growth factor (EGF) was purchased from R&D Systems (cat.: 236-EG) and reconstituted in sterile phosphate-buffered saline (PBS). Cells were serum-deprived for approximately 16 h prior to stimulation with 50 ng ml^−1^ EGF for the indicated durations. Imatinib mesylate (Gleevec) was obtained from LC Laboratories (cat.: I-5508) and reconstituted at 10 mM stock in dimethyl sulfoxide (DMSO). Cells were incubated with 1 µM Imatinib (or equivalent volume of DMSO) for 4 h (U2OS cells) or 2 h (K-562 cells) in serum-free DMEM prior to cell lysis. SHP099 (hydrochloride) was purchased from MedChem Express (cat.: HY-100388A) and reconstituted at 10 mM in DMSO. Cells were incubated with the indicated concentration of SHP099 (or equivalent volume of DMSO) in serum-free DMEM for 2 h prior to cell lysis. MLN4924 was from Active Biochem (cat.: A-1139) and was reconstituted at 1 mM in DMSO.

### 4.5. SDS-PAGE and western blotting

Reduced protein extracts (typically 10–20 µg protein unless stated otherwise) or immunoprecipitates (IPs) were separated on 8% SDS-PAGE gels, or NuPAGE 4–12% Bis-Tris protein gels (Invitrogen) by electrophoresis. Subsequently, proteins were transferred onto polyvinylidene fluoride (PVDF) (Millipore) or nitrocellulose membranes (Amersham), prior to blocking in 5% (w/v) non-fat milk (Marvel) in TBS-T (50 mM Tris–HCl pH 7.5, 150 mM NaCl, 0.2% Tween-20). Membranes were incubated overnight at 4°C in 5% (w/v) bovine serum albumin (BSA)-TBS-T or 5% (w/v) milk-TBS-T with the appropriate primary antibodies. Membranes were subsequently washed in TBS-T and incubated with HRP-conjugated secondary antibodies in 5% (w/v) milk-TBS-T for 1 h at RT. Membranes were subjected to further washing in TBS-T, prior to detection using enhanced chemiluminescence (ECL) reagent (Thermo Scientific) and exposure on medical X-ray films (Konica Minolta) as described previously [12,41].

### 4.6. RNA-isolation, cDNA synthesis and RT-PCR

Cells were washed in PBS and harvested using standard procedures. RNA was isolated following the manufacturer's guidelines (Qiagen). cDNA synthesis and RT-PCR were performed as described previously [39,42]. SHP2 (forward: GACGGCAAGTCTAAAGTGAC; reverse: GCTTTCTATTTCAGCAGCAT) and GAPDH (forward: TGCACCACCAACTGCTTAGC; reverse: GGCATGGACTGTGGTCATGAG) primers were from Invitrogen. Statistical analysis was performed using Student's *t*-test on data obtained from three independent biological replicates. A *p*-value of less than 0.05 was deemed significant.

### 4.7. siRNA transfection

Cells were transfected with 20 nM (final) small interfering RNA (siRNA) oligonucleotides using 4 µl Lipofectamine RNAiMAX (Invitrogen) transfection reagent per well in a 6-well cell culture plate, in a final volume of 1 ml Optimem (Gibco). Cells were lysed at 8 h, 24 h and 48 h post-transfection. For the 24 and 48 h time points, the transfection medium was replaced by fresh growth medium after 16 h post-transfection.

## Supplementary Material

Electronic supplementary figures and figure legends
