# Supplementary material for: Targeting endogenous proteins for degradation through the affinity-directed protein missile system
Source: Open Biol. 2017 May 10;7(5):170066. doi: 10.1098/rsob.170066 (PMC5451546; doi:10.1098/rsob.170066)
Supplement: Electronic supplementary figures and figure legends [file rsob170066supp1.pdf]

Targeting endogenous proteins for degradation through the affinity-directed protein missile system.

Luke J. Fulcher, Luke D. Hutchinson, Thomas J. Macartney, Craig Turnbull and Gopal P. Sapkota*

Electronic Supplementary Material figure legends and figures:

figure S1: Effects of SHP2 AdPROM on SHP2 mRNA levels. MDA-MB-468 cells infected with VHL, aCS3 or VHL-aCS3 retroviruses were lysed and mRNA extracted. Uninfected wild-type (WT) cells were included as controls. Complementary DNA was generated and the q-RTPCR performed to measure SHP2 mRNA transcript levels across the different conditions. SHP2 transcript levels were normalised to GAPDH transcripts. NS and * refer to comparison between VHL, aCS3 or VHL-aCS3 and WT. NS = non-significant. *= $P<0.05$; N=3.

figure S2: Proteolytic AdPROM employs CUL2-CRL for SHP2 destruction (NSa1). ***(a)*** Uninfected U2OS cells (WT) or cells infected with retroviruses encoding FLAG-VHL-aNSa1 or control viruses (VHL alone, FLAG-aNSa1 alone) were treated with MLN4924 (1 µM) for 0, 6 or 24 h. Cells were lysed and extracts (20 µg protein) were resolved by SDS-PAGE and transferred to PVDF membranes, which were subjected to Western blotting with the indicated antibodies. HIF1α serves as a positive control for MLN4924 treatment. ***(b)*** U2OS cells treated with or without MLN4924 (1 µM) for 24 h were lysed and extracts (1 mg protein) subjected to immunoprecipitation (IP) with anti-FLAG M2 resins. Following elution, both IPs and input extracts (20 µg protein) were resolved by SDS-PAGE and transferred to PVDF membranes, which were subjected to Western blotting with the indicated antibodies.

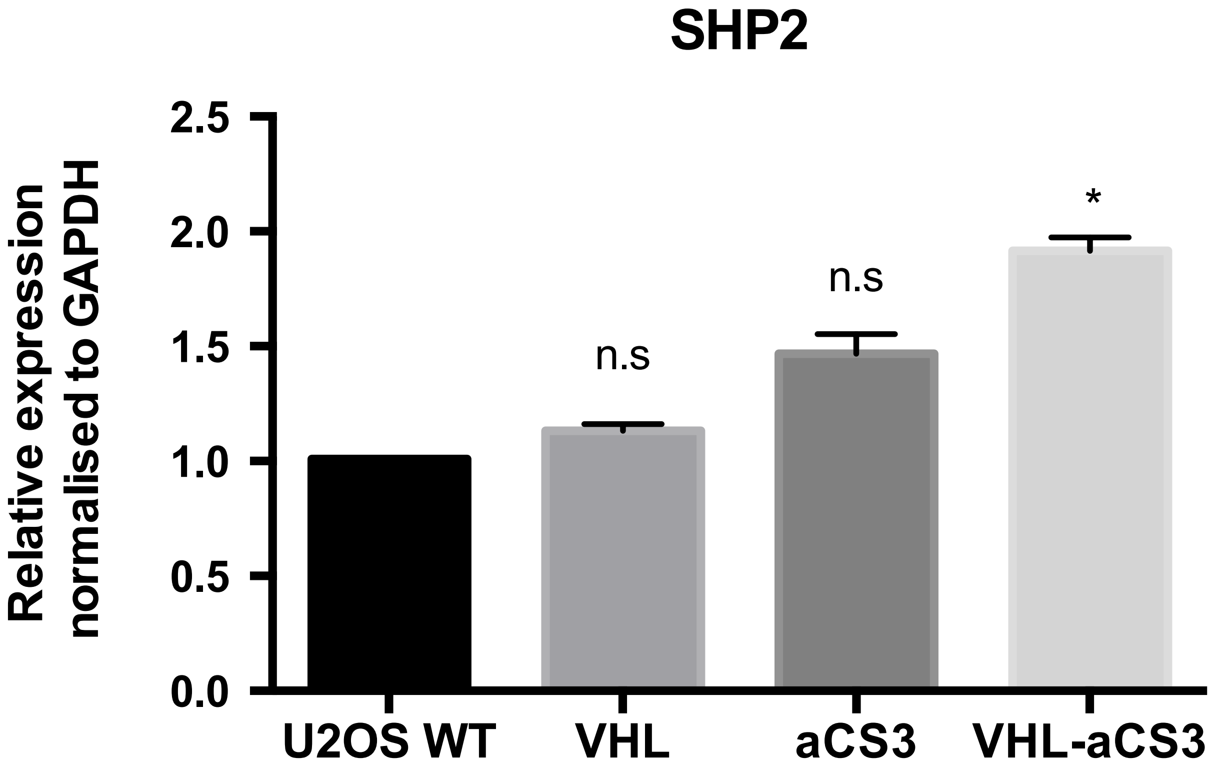

Fulcher et al, electronic supplementary material Figure S1

(a)

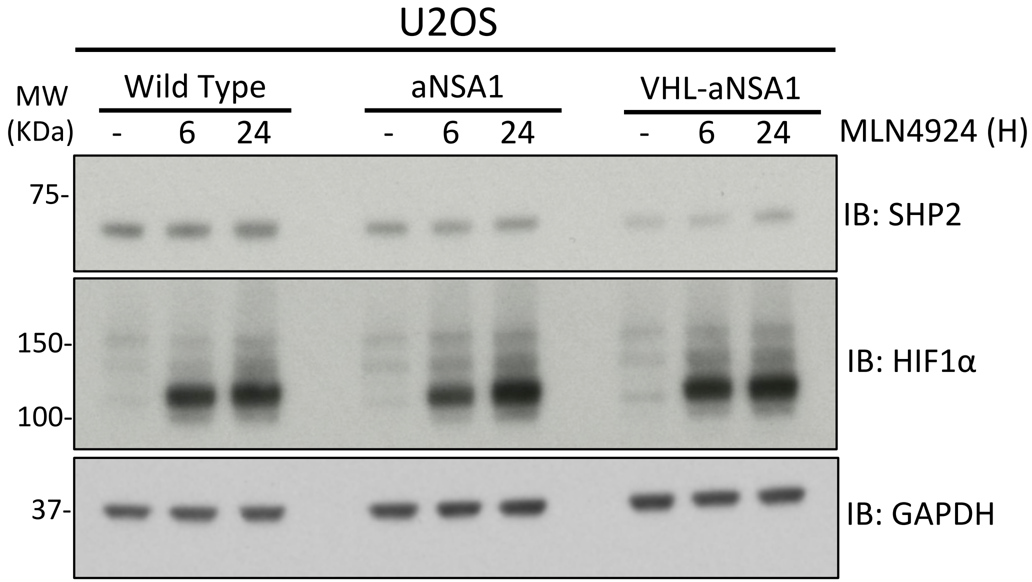

(b)

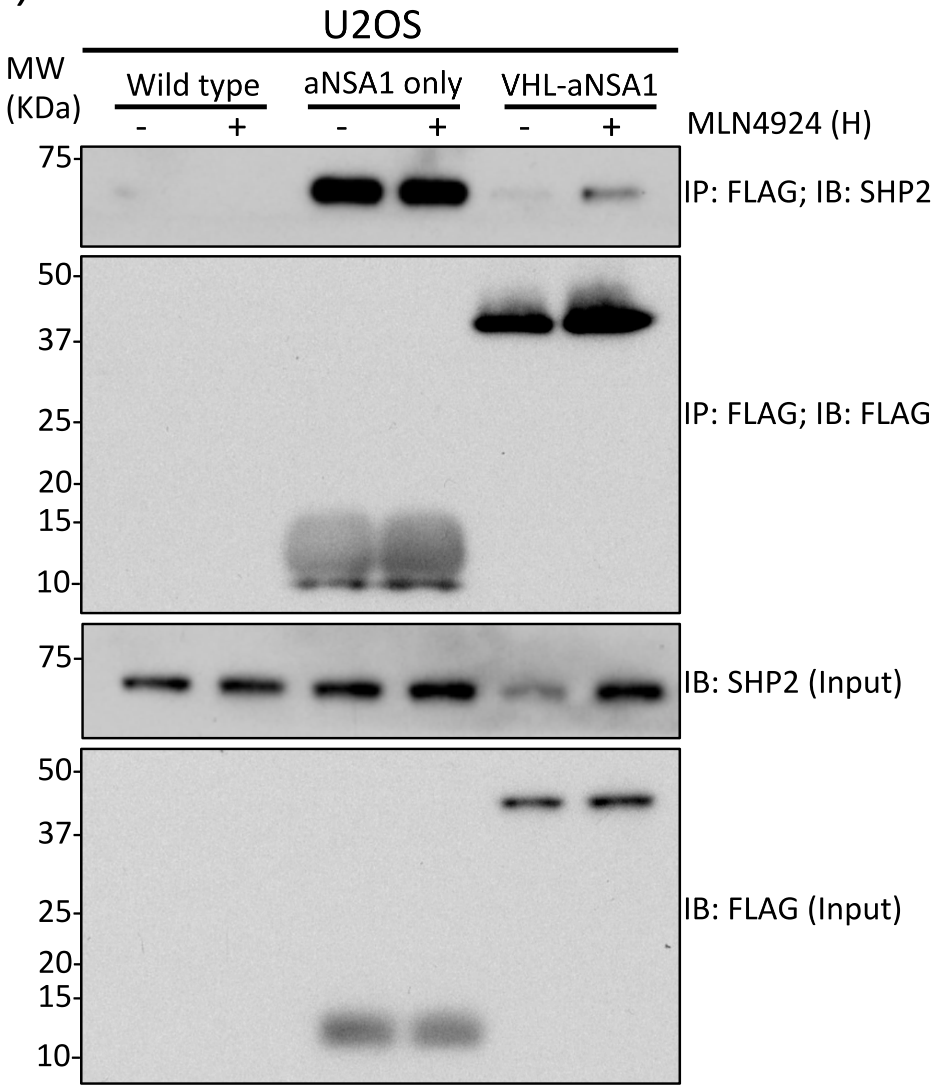

Fulcher et al, electronic supplementary material Figure S2
